# Taking a break: The effect of taking a vacation from Facebook and Instagram on subjective well-being

**DOI:** 10.1371/journal.pone.0217743

**Published:** 2019-06-06

**Authors:** Sarah M. Hanley, Susan E. Watt, William Coventry

**Affiliations:** School of Psychology and Behavioural Sciences, University of New England, Armidale, NSW, Australia; West Pomeranian University of Technology, POLAND

## Abstract

Social Networking Sites (SNS) such as Facebook and Instagram have relocated a large portion of people’s social lives online, but can be intrusive and create social disturbances. Many people therefore consider taking an “SNS vacation.” We investigated the effects of a one-week vacation from both Facebook and Instagram on subjective well-being, and whether this would vary for passive or active SNS users. Usage amount was measured objectively, using RescueTime software, to circumvent issues of self-report. Usage style was identified at pre-test, and SNS users with a more active or more passive usage style were assigned in equal numbers to the conditions of one-week SNS vacation (*n* = 40) or no SNS vacation (*n* = 38). Subjective well-being (life satisfaction, positive affect, and negative affect) was measured before and after the vacation period. At pre-test, more active SNS use was found to correlate positively with life satisfaction and positive affect, whereas more passive SNS use correlated positively with life satisfaction, but not positive affect. Surprisingly, at post-test the SNS vacation resulted in lower positive affect for active users and had no significant effects for passive users. This result is contrary to popular expectation, and indicates that SNS usage can be beneficial for active users. We suggest that SNS users should be educated in the benefits of an active usage style and that future research should consider the possibility of SNS addiction among more active users.

## Introduction

Taking a vacation from social networking sites (SNS) such as Facebook and Instagram is a relatively new phenomenon, whereby people disconnect from one or all of their SNS for a period of time. Research has found that SNS usage has many benefits, mainly through increasing one’s social capital which positively affects self-esteem and subjective well-being (SWB) [1, 2], but it can also be detrimental to SWB [3–5]. Prior research has shown that taking a break from SNS is often motivated by social disturbances such as feeling bad from upward social comparison, exposure to distorted (overly positive) presentation, feeling meaningless or bored, and interpersonal quarrels [6–11]. However, when people take an SNS vacation, they separate themselves not only from the negative effects of SNS usage but also from its benefits. This raises the question of whether taking an SNS break has positive or negative effects on subjective well-being.

Subjective well-being resides within the experience of the individual and has two components: affective well-being (positive and negative affect) and life satisfaction [12–13]. Research has found that the way in which people engage with SNS, whether it is active or passive, is a key variable in how SNS usage affects SWB [14]. ‘Active usage’ involves creating content and communicating directly with others; for example, posting status updates, commenting, chatting and sharing posts [3]. Conversely, ‘passive usage’ involves consuming other people’s information without communicating with others [5]. Passive activities include browsing newsfeeds, following others communications, examining friends’ profiles, and looking at their photos without responding [5]. Active and passive usage are not completely distinct constructs, and research has found that they moderately correlate because active users must also consume other people’s information while engaging with SNS [15]. We refer to ‘active users’ and ‘passive users’ to reflect people who tend toward a more active or passive usage style along a continuum from purely passive to predominantly active usage.

Research on SNS and social well-being by Burke et al. [16] and Ellison et al. [1] concluded that active usage is associated with the formation and maintenance of social capital, which relates to positive consequences of increased self-esteem and subjective well-being. In contrast, passive usage relates to decreased SWB [3–5]. Most people tend to only post positive things about their life developments on SNS [5], creating an unrealistic presentation of self. When passive users consume this information, they engage in what is known as ‘upward social comparison,’ and conclude that others are happier and better off than themselves [17–18]. This can provoke envy, depression and reduced SWB [3, 5, 19–20], an effect which is stronger among people who are more prone to social comparison [21–23].

If passive usage relates to decreased subjective well-being, then disengaging from this online behaviour may improve levels of subjective well-being. However, few studies have examined whether an SNS vacation reduces these negative consequences, and produced mixed results. Hinsch and Sheldon [24] conducted two studies that examined the effects of reducing (Study 1) or ceasing (Study 2) Facebook or online gaming for 48 hours. Both studies found that reducing or ceasing Facebook usage/online gaming increased participants’ life satisfaction, but decreased positive affect. Tromholt [25] used a large sample and a Facebook break of one week. This study found increases in life satisfaction and positive affect in the treatment group (Facebook break) when compared with the control group (no Facebook break). The effects were stronger among heavy Facebook users, passive users, and those who tended to envy others. Conversely, Vanman, Baker, and Tobin [26] found cortisol levels in experimental group participants were reduced after the Facebook break, suggesting that Facebook is stressful. This was more so when passive usage was low; there was no moderation effect of active usage. The experimental group participants also experienced reduced satisfaction with life, when compared with the control group (whose life satisfaction increased during that period).

These studies shared a common limitation: SNS usage and reduction in usage were measured using self-report which can be inaccurate or prone to bias due to demand characteristics [27]. People are often unaware of how frequently they check or how much time they spend on SNS and would have difficulty reporting accurate usage. There was no mechanism to check Facebook usage had reduced or ceased during the experiments other than self-report.

The current research aimed to address the limitations of the existing research and to provide a more definitive answer to the question of the effects of an SNS vacation on subjective well-being. Using an experimental design, we tested the effect of having a more complete break from SNS (Facebook and Instagram together) on subjective well-being, taking into account active or passive usage styles. Importantly, we used an objective measure of SNS usage using software called ‘RescueTime’ which was installed on their mobile and laptop devices. Based on pre-test measures, participants were categorized as more active or more passive users and were then randomly allocated to an SNS vacation or waitlist condition. In the SNS vacation condition, access to Facebook and Instagram were blocked on registered devices for one week, and any usage from other devices could be identified.

Because passive usage is associated with higher upward social comparison [22] and lower SWB [4–5, 15], we expected an SNS vacation would benefit passive users, resulting in an increase in overall life satisfaction and affective well-being. Conversely, because active users reap benefits from using SNS, such as social capital and self-esteem, we expected disconnecting for a week could be counterproductive. Consistent with previous research, we measured two different components of subjective well-being: life satisfaction, and affective well-being (positive and negative affect). We hypothesized that there would be a moderating effect of usage style such that, after the SNS vacation, life satisfaction and affective well-being would be improved among more passive users, and reduced among more active users.

Our study also included a correlational component, which tested whether, at pre-test, the frequency of SNS usage (minutes) and passive and active usage correlated with life satisfaction and affective well-being. It was hypothesized (1) that more frequent SNS usage (minutes) would relate negatively to life satisfaction and affective well-being; (2) that passive usage would relate negatively to life satisfaction and positive affect; and (3) that active usage would relate positively to life satisfaction and positive affect.

## Materials and methods

### Participants

Seventy-eight participants completed the study; comprising 35 males (*M* = 29.49, *SD* = 5.61) and 43 females (*M* = 31.95, *SD* = 8.05) ranging from 18 to 48 years old (*M* = 30.85, *SD* = 7.12). Recruitment was restricted to this age range since SNS usage (particularly Instagram) is appreciably lower in older individuals [28–31]. The participants were recruited using Prolific Academic (an online research participant pool; 66 participants), and Facebook pages associated with the University of New England, Australia (12 participants). To establish a broad sample the study was opened to the English-speaking countries that had a large population of SNS users, based on country comparisons [32–33], namely Australia, the United Kingdom and the United States of America, recruiting *n* = 24, 33 and 21 from each of these countries respectively. No differences were observed by country, age or gender for the life satisfaction, positive affect, negative affect or active usage score variables (all *p* > .05). Participants were paid £3 upon completion of the two-week study. About half the participants did not use their Instagram account regularly (*n* = 40); Facebook was the more popular SNS. The data was collected in late 2016.

There was some attrition between the phases. One hundred and nine participants completed Phase 1 and installed RescueTime on their phone. Of these, ninety-seven completed the remaining phases. However, RescueTime detected 19 who did not fully comply with the SNS vacation and had to be excluded from the dataset leaving a final sample of 78 (40 experimental, 38 control) who fully completed the study. There were 19 males and 19 females in the control condition, and 16 males and 24 females in the experimental condition.

### Materials

#### RescueTime

While prior studies relied on self-report measures of Facebook usage, this study used software called *RescueTime* (available from https://www.rescuetime.com/), an application which monitors log-ins, time spent on SNS (minutes), and blocks SNS on devices. This ensured more accurate, unbiased measures of usage than in previous studies, and allowed us to monitor compliance in the ‘vacation’ condition. Instagram and Facebook usage were combined to create a variable called frequency of SNS usage (minutes). RescueTime was downloaded onto all devices (including mobile phones, laptops and tablets) where participants frequently used SNS. The application was not available on iPhone, so participants were required to have an Android phone.

#### Life satisfaction

Life satisfaction was measured using the Quality of Life Enjoyment and Satisfaction Questionnaire–18 (Q-LES-Q-18) [34]. To address issues of demand characteristics, half the items were used at pre-test and the other half at post-test [27]. The questionnaire was split in half by matching factor loadings of approximately equal questions from each domain. This scale assesses four domains of life enjoyment and satisfaction over the past week–physical health, subjective feelings, leisure and time activities, and social relationships. The final question “How satisfied have you been with medication?” was excluded as it was not applicable to this study. Responses were scored on a scale from 1 = “Not at all or never” to 5 = “Frequently or all the time” and an average score was computed from the items. The split half-reliabilities were α = .93 and α = .85.

#### Positive and negative affect

Positive affect (PA) and negative affect (NA) were measured using the Positive and Negative Affect Schedule (PANAS; Watson et al. [35]). Given that this scale was made up of subscales, split-half was not conducted; instead, items were presented in random order to combat learning effects. The PA and NA scales each comprise ten emotive items, such as “excited” (PA) and “afraid” (NA). Individuals indicated on a scale from 1 = “Very slightly / not at all” to 5 = “Extremely” the extent to which they experienced each of these emotions in the past week. PA and NA scores could range from 10–50, with higher scores signifying higher PA or NA. Cronbach’s alphas for the PA and NA were .93 and .87 in this study, demonstrating high internal consistency.

#### Passive and Active Usage Scale

The current research needed to measure passive and active usage in Facebook and Instagram combined. No such scale existed, so it was necessary to create a measure specifically for this study. Eighteen items, rated from 1 = “Never” to 5 = “Frequently,” were created. These were based on Pagani et al.’s scale [36] for the active usage items (e.g., “Meet new people / make new friends”), and Verduyn et al. [3] for the passive items (e.g., “Scroll through my newsfeed”), and reflected the sorts of activities users of Facebook and Instagram might engage in.

A pilot study was conducted to determine the factor structure before use. We expected to find two factors reflecting the active and passive sub-scales. In the pilot study, 230 Australian residents ranging from 18–48 years old (*M* = 29.63, *SD* = 7.28) rated the preliminary set of 18 items (Table 1) as an online survey. Principal component analysis with direct oblimin rotation assessed the underlying factor structure. Two factors had eigenvalues greater than one (Table 1). We labeled these “Active” and “Passive” to reflect the type of usage. Five items were removed: when using a cutoff of .45 they loaded on either both factors or neither factors. This left 13 items, with six in the Passive sub-scale and seven in the Active. The internal consistency of the subscales was reliable, α = .82 (Active) and α = .80 (Passive). The current study found similar reliability on the two sub-scales, α = .82 (Active) and α = .87 (Passive).

**Table 1:** Factor loadings based on a principal components analysis with oblimin rotation for 18 items from the Passive and Active Usage Scale (PAUS) (N = 230). Asterisked items were included in the final scale.

Facebook	Factor 1: Active	Factor 2: Passive
*Scroll through my newsfeed		**-0.75**
Chat to friends on messenger	0.36	
View other people's profiles	0.34	-0.30
Watch videos	0.34	
*Comment on people's posts	**0.59**	
*View other people's posts and status updates		**-0.55**
*Write status updates or post photos / videos of my own	**0.71**	
*Create invitations or organise social gatherings with my friends	**0.72**	
*Meet new people / make new friends	**0.85**	
Click on people's profiles that I don't know	0.42	
**Instagram**		
*Scroll through my newsfeed		**-0.85**
*Look at other people's images		**-0.88**
*Contact friends via DM (direct message)	**0.65**	
Look at celebrity pages / fitness pages	0.35	-0.38
*Comment on friends / people's images	**0.57**	
*Like people's / friend’s images	0.35	**-0.56**
*Post my own photos	**0.46**	
*Click on profiles that you don't follow and view their images		**0.46**

* Included in final scale.

Each participant’s average response to the passive and active sub-scales were averaged, producing an active usage score and a passive usage score from 1–5. To reflect a continuum from passive to active usage, a single continuous measure was then created by subtracting the scores on the Passive sub-scale from those on the Active sub-scale. This gave each participant an ‘active user score’ (AUS) from -4 to 4, with higher results indicating more active usage compared with passive usage. This technique has been employed elsewhere: for instance, in research involving subjective well-being, with the scores on negative affect being subtracted from positive affect to optimally differentiate subjects on a single scale of positive and negative affect [21, 36]. We called the scale the Passive and Active Usage Scale (PAUS). Thus, from the PAUS scale we had an active usage score, a passive usage score, and an active user score (AUS).

#### Procedure

The study was conducted with approval of the University of New England’s Human Research Ethics Committee—Approval No HE16-086, Valid to 05/05/2017. The study was advertised to appeal to participants who wanted to take a short break from Facebook and Instagram. Consent was obtained via an anonymous online survey which was created using Qualtrics software. After providing consent, participants indicated their age, gender, country of residence and whether they had an Android Smartphone. They were also asked to indicate all of the devices they currently used to access SNS. They then proceeded to the PAUS, followed by instructions on installing the RescueTime application onto their Android phone and other devices. The researchers cross-checked to see that RescueTime had been installed on all devices that the participants indicated in the first survey. Participants were then instructed to use SNS normally for one week (this established baseline SNS usage). After the monitoring week was complete, participants received a link to the second online survey.

Participants were then rank ordered on the AUS dimension and, starting from the highest score and working down, every 2^nd^ individual was assigned to the experiment condition and all others to the control condition, thus ensuring these groups were equivalent on the AUS. The experimental group were blocked from SNS for one week and asked to temporarily remove Facebook and Instagram applications from their phones, whereas those in the control condition were told they could continue to use SNS normally and would have the opportunity to take their SNS vacation at a later date. Any SNS use on registered devices during this time was detected with the RescueTime application. Participants completed the post-test survey at the end of the vacation period.

#### Analyses

Correlations were computed to test the hypothesized relationships between SNS usage amount, usage style, life satisfaction and affective well-being. Then, moderations were conducted to test the effects of the SNS vacation , an IV, on life satisfaction and affective well-being, the DVs, which we expected would be improved among individuals with a low AUS (more passive users) and reduced among those with a higher AUS (more active users). More precisely, the DVs were the changes from pre-test (T1) to post-test (T2), computed by subtracting the score at T1 from that at T2, which was done for the three DVs, life satisfaction, positive affect, and negative affect, with separate moderations run for each. The small sample size could not accommodate two moderators, so we used the composite AUS as a moderator, rather than including active and passive usage as separate moderators. Hence, the IVs of both moderations were (a) being in the experiment or control condition for an SNS vacation (condition), (b) AUS, and (c) AUS × condition. In addition, gender and SNS usage at baseline were included as control variables.

## Results

RescueTime recorded, on average, 449 minutes (*SD =* 43.6) of SNS usage during the baseline monitoring week, with a range from 3 to 1664 minutes. The distribution was positively skewed; median usage was 192 minutes (mode = 5.6). SNS usage at baseline did not differ significantly between the Experimental and Control groups (*t*_*log-transformed SNS usage amount*_ = -.41, *p* = .69).

The results of the correlations, presented in Table 2, show that the amount of time spent on SNS did not significantly correlate with life satisfaction or affective well-being (PA and NA). Active usage correlated positively with positive affect and life satisfaction. Passive usage correlated positively (but weakly) with life satisfaction, but not with PA or NA. A paired sample *t*-test revealed that, on average, participants engaged in more passive usage (*M* = 3.05, *SD =* .98) than active usage (*M* = 2.25, *SD* = .87), *t*(77) = -8.45, *p* < .001.

**Table 2:** Correlation matrix between active and passive usage and SWB (N = 78).

Measure	*M*	*SD*	1	2	3	4	5
**1. Active usage** **2. Passive usage**	2.25 3.05	.87 .98	- .601**	-			
**3. Positive Affect**	29.74	7.93	.354**	.183	-		
**4. Negative Affect**	20.26	8.19	-.041	.043	-.035	-	
**5. Life Satisfaction**	27.51	5.31	.205*	.189*	.736**	-.112	-
**6. Time on SNS (weekly, log-transformed)**	2.18	.64	-.038	-.030	-.021	-.007	-.106
**7. Gender (1 = Male, 2 = Female)**	1.55	.50	.143	.192	.062	.025	.053

Note. Correlations are significant

* *p* < .05 (one-tailed)

** *p* < .01 (one-tailed).

**Fig 1:**
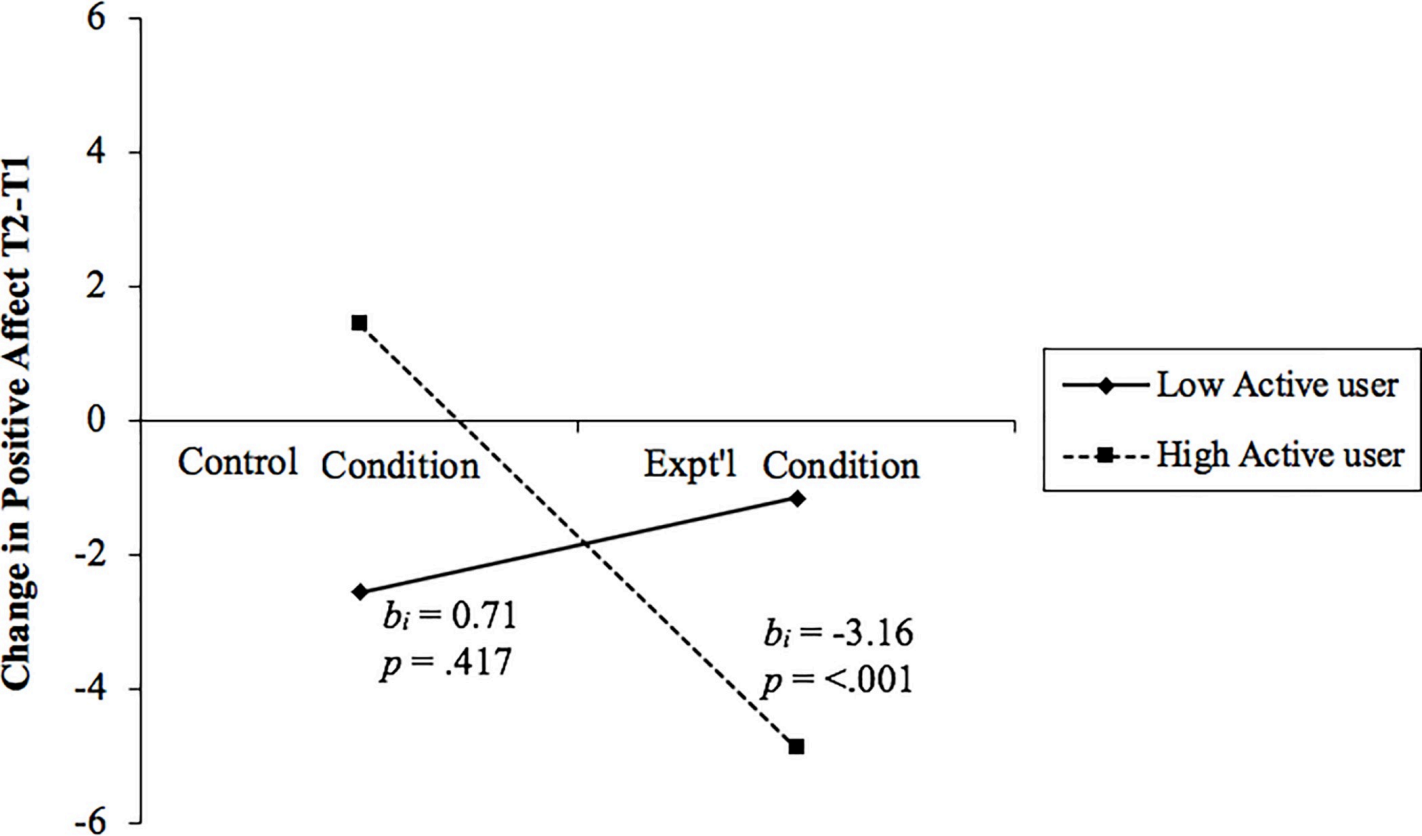
Moderation effect of active user score on the effect of experimental condition on change in positive affect from T1 to T2. Positive scores indicate an increase in T2, negative scores indicate a decrease. The unstandardized betas (b_i_) and significance (p) are reported, adjacent to each line, for the simple slopes analysis of the interaction.

**Fig 2:**
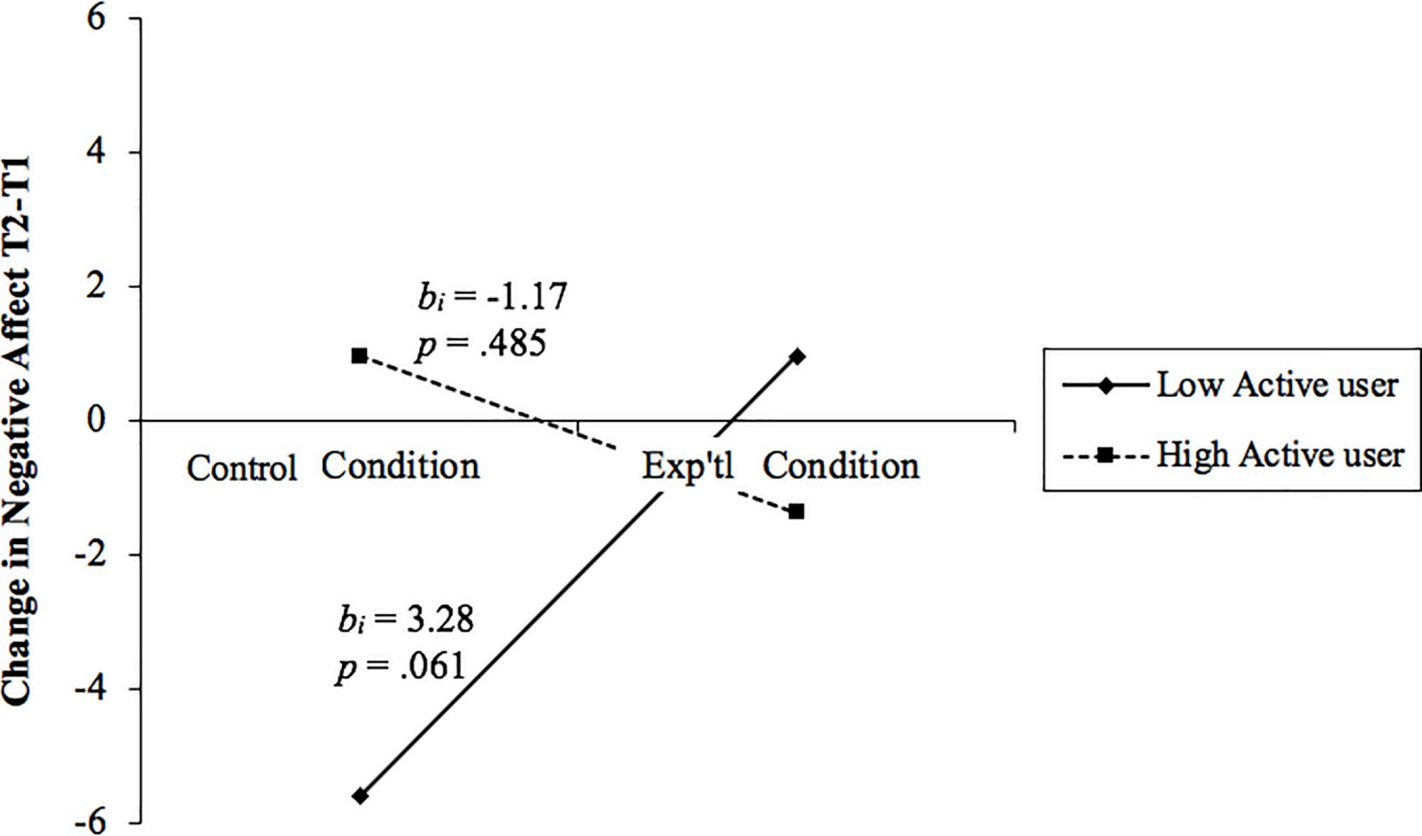
Marginally significant moderation effect of active user score on the effect of experimental condition on change in negative affect from T1 to T2. Positive scores indicate an increase in T2, negative scores indicate a decrease. The unstandardized betas (b_i_) and significance (p) are reported, adjacent to each line, for the simple slopes analysis of the interaction.

The results (Table 3) revealed a significant interaction of experimental condition and usage style on PA and a marginally significant interaction of experimental condition and usage style on NA (*p =* .07). There were no significant effects on life satisfaction. Breaking down the interaction effect on PA, the biggest change was observed in the experimental condition, such that PA decreased from T1 to T2 for more active users, opposite to that hypothesized, and showed little change for more passive users (Fig 1), where we hypothesized a decrease. There was little change in PA for control group participants. Simple slopes analysis (Figs 1 and 2) revealed a significant negative relationship between condition (control vs experimental) and PA change for more active users. For the more passive users, there was no significant effect of the SNS vacation on positive affect change.

**Table 3:** Multiple regression models examining experimental condition, SNS usage style, and their interaction as predictors of change in positive affect (PA), negative affect (NA), and life satisfaction from Time 1 to Time 2. Standardized coefficients are presented (N = 78).

Predictor	Model 1 PA Change	Model 2 NA Change	Model 3 Life Sat. Change
**Experimental condition (Control = 1, Experimental = 2)**	-.16	.10	.05
**Gender**	-.10	-.12	.11
**Time on SNS at baseline (log)**	-0.05	-.01	-.11
**Usage style (active user score centered, high scores = more active usage)**	.009	.10	-.04
Condition*Usage style	-.25 (*p =* .03)*	-.22 (*p =* .*07)*	-.02
***R***	.30	.25	.17
***F***	1.49	.99	.40

* *p <* .*05*

There was a similar interaction effect on NA. For more passive users, NA decreased in the control group and increased in the experimental group (Fig 2). However, the simple slope was only marginally significant (*p* = .06). For active users, NA showed little change in either condition.

## Discussion

Previous studies found that active SNS usage related to increased PA and life satisfaction (subjective well-being) whereas passive usage and more frequent usage related to decreased PA and life satisfaction (see Verduyn [14] for a review). Based on this, people who engage in mainly passive SNS usage could be expected to benefit from an SNS vacation, but people with a more active usage style would not. We tested the effects of a one-week vacation from Facebook and Instagram together, to provide a more complete SNS vacation than taking a break from just one SNS alone. We also circumvented issues of self-report by using software to monitor and block Facebook and Instagram usage, and controlled social desirability effects in reporting life satisfaction by using different questions at pre- and post-test. Participants were recruited from three different countries, and so the findings are not confined to just one national context.

The results revealed a moderation effect of usage style, such that taking a vacation from Facebook and Instagram decreased PA for more active users, and not for more passive users. There was also a small effect on NA, such that NA improved for passive users in the control group, and not the experimental group. There were no significant effects on life satisfaction.

Like the current study, Hinsch and Sheldon [24] found that an SNS break (Facebook and online gaming) resulted in decreased PA. This was not found by Vanman et al. [26], nor by Tromholt [25]. In the current results, decreased PA resulting from the SNS break was restricted to more active SNS users. Active users build and maintain social capital and consequently increase their self-esteem and SWB through SNS use [1, 16], therefore it is an integral part of their lives. Hence, they most likely depend on SNS to maintain and develop their social ties, which may explain the decrease in PA in this study. As such, highly active users may have a level of dependency on SNS. Hormes, Kearns, and Timko [37] found evidence of disordered use of SNS among 9.7% of an American university cohort. If this is elevated among active SNS users, the proportion of addicted active users could be quite high. We believe this is an important direction for future research. This effect was also visible in the overall positive correlations between active usage and life satisfaction and PA.

Passive users in the control group experienced slightly decreased NA at T2 compared with those in the experimental group. However, this was only marginally significant. Vanman et al. [26] analyzed participants’ thoughts on being allocated to an SNS vacation, and many showed dread at this prospect. It is possible that our control group participants were relieved at being allocated to this condition, and felt less negativity in their SNS use during the following week as a result. It could also be argued that, as they were placed on a waitlist to experience the SNS vacation, this may have had the effect of making SNS more valued during the interim, decreasing NA.

Time spent on SNS did not correlate with any of the T1 measures of SWB (PA, NA, or life satisfaction). This is an interesting result, as ours was the first study to measure time spent on SNS objectively and to correlate it with subjective well-being. Passive usage also showed little relationship with T1 subjective well-being, with no relationships with PA or NA, and only a small anomalous relationship with life satisfaction. Wang et al. [22] found the same effect in a Chinese study of passive SNS usage. In their research, passive usage exerted an indirect effect on subjective well-being, which was mediated by upward social comparison and self-esteem, and moderated by participants’ tendency to engage in social comparison. Ding et al. [20] reported similar results, where envy (a product of upward social comparison) mediated an association between passive SNS use and low subjective well-being, and this was stronger among females than males. Tromholt [25] found that there was more benefit of a Facebook vacation when Facebook envy was high. The current research included the Facebook Envy Scale [38], so as a posthoc analysis we checked the possibility that envy mediated the relationship between passive usage and subjective well-being. While envy correlated negatively with positive affect (*r* = -.42) and life satisfaction (*r* = -.48), it did not correlate with passive usage. Hence, no indirect effect was present. Wang et al.’s [22] results raise interesting possibilities for the current research and suggest that a more fine-grained picture could be obtained by including measures of upward social comparison, social comparison tendency, and self-esteem.

Given the worldwide popularity of SNS, research on their relationship with SWB has important implications for the general public. The clinical implications of this research are that users who engaged actively, posted their own content, and socialized on SNS were more positive than passive users. Additionally, active usage was positively correlated with life satisfaction and positive affect. Those who scored higher in active usage experienced a decrease in positive affect when they took a vacation from SNS, indicating a causal effect of active SNS usage on positive affect. Therefore, active usage seems to be the most beneficial way to engage with SNS in terms of positive affect. A potential intervention might be to educate passive users on the benefits of active usage, the negative consequences of passive use and ways to improve their positive experience on SNS. While usage type may depend on other variables (e.g., personality), passive users could at a minimum gain more positive experience from commenting on friends’ posts and engaging with friends via messages.

### Limitations

There were several limitations to this research. Participants volunteered because they wanted to take a break from SNS. This improved the ecological validity of the study, as people would usually take an SNS break voluntarily. However, it also created a possibility of self-selection effects. For example, our participants may have been high in self-monitoring propensity, meaning that they could have a personality characteristic(s) that is different from the general population. The current results will generalize best to similar situations, where people choose to take a break from SNS. Having said this, Hinsch and Sheldon [24] found similar effects in their two studies, one of which used self-selected volunteers, the other of which allocated participants to condition as part of their course requirements. Thus, self-selection (or not) does not appear to be of critical importance in the research design.

The current study observed no changes in life satisfaction from T1 to T2. Previous researchers used the five-item Satisfaction with Life Scale [12] and presented it at each phase of the study. To avoid demand effects from presenting the same items repeatedly, we measured life satisfaction with the Q-LES-Q-18, using half the items at T1 and the other half at T2. It is possible that the different results for life satisfaction in the current study arose in the choice of a different scale, or perhaps by using half the items at a time. Perhaps the demand effects in previous studies were more transparent than in the current study, leading to results that were more consistent with experimenter expectation.

The final sample was relatively small, and it is likely that more effects would be found with a larger sample. The fact that participants had to install RescueTime on their devices seems to have been a barrier to participation, and it is possible that participants who completed the study may have been particularly conscientious or determined.

Despite these limitations, the current research has demonstrated that, among people who would like to take an SNS vacation, more active SNS users are likely to experience decreased positive affect when they take an SNS vacation, indicating a causal relation between active SNS use and positive affect, while more passive SNS users are unlikely to gain a direct benefit. This has many interesting implications, including the extent to which active users may be more prone to SNS addiction. For passive users, an SNS vacation may not be the best way forward. Future research could investigate the effects of targeting highly passive users with an intervention on how to use SNS actively. Alternatively, it could include measures of social comparison to deduce how this is related to subjective well-being, and whether those who engage in social comparison more experience an increase in SWB after an SNS vacation.

Nineteen participants did not fully comply with the SNS vacation, despite the assistance of RescueTime; fortunately RescueTime could detect this. This is an interesting group, as they may have experienced particularly strong negative responses to separation from SNS. Future research could examine the profile (active or passive) of users who failed to comply with the vacation and whether this is related to SNS addiction or excessive usage. It would be worthwhile investigating whether the finding that active users became less positive could be due to more propensity to SNS addiction among highly active users.

## Conclusions

In conclusion, the present study confirmed that active SNS usage is positively related to SWB. Furthermore, the predicted negative relationships with passive usage and SWB were not found. In fact, taking a vacation from SNS for a week was detrimental to more active users’ positive affect, and it did not decrease negative affect or improve life satisfaction. This result is contrary to much popular expectation, and indicates that SNS usage can be beneficial for active users. We suggest that users might be educated on the benefits of active usage, and on ways to improve their positive experience on SNS. We also suggest that this finding is investigated further to assess whether highly active SNS users may experience decreased positivity due to SNS addiction.

## References

[1] Ellison NB, Steinfield C, Lampe C. The benefits of Facebook “friends:” Social capital and college students’ use of online social network sites. Journal of Computer‐Mediated Communication. 2007 7;12(4):1143–68. 10.1111/j.1083-6101.2007.00367.x

[2] Valenzuela S, Park N, Kee KF. Is there social capital in a social network site?: Facebook use and college students' life satisfaction, trust, and participation. Journal of computer-mediated communication. 2009 7 1;14(4):875–901.

[3] Verduyn P, Lee DS, Park J, Shablack H, Orvell A, Bayer J, Ybarra O, Jonides J, Kross E. Passive Facebook usage undermines affective well-being: Experimental and longitudinal evidence. Journal of Experimental Psychology: General. 2015 4;144(2):480 10.1037/xge0000057 25706656

[4] Sagioglou C, Greitemeyer T. Facebook’s emotional consequences: Why Facebook causes a decrease in mood and why people still use it. Computers in Human Behavior. 2014 6 1;35:359–63. 10.1016/j.chb.2014.03.003

[5] Krasnova H, Wenninger H, Widjaja T, Buxmann P. Envy on Facebook: A hidden threat to users’ life satisfaction? 1477–1491. 11th International Conference on Wirtschaftsinformatik, 27th February– 01st March 2013, Leipzig, Germany

[6] Chou HT, Edge N. “They are happier and having better lives than I am”: the impact of using Facebook on perceptions of others' lives. Cyberpsychology, Behavior, and Social Networking. 2012 2 1;15(2):117–21. 10.1089/cyber.2011.0324 22165917

[7] Lee SY. How do people compare themselves with others on social network sites?: The case of Facebook. Computers in Human Behavior. 2014 3 1;32:253–60.

[8] Haferkamp N, Krämer NC. Social comparison 2.0: Examining the effects of online profiles on social-networking sites. Cyberpsychology, Behavior, and Social Networking. 2011 5 1;14(5):309–14. 10.1089/cyber.2010.0120 21117976

[9] Cho IH. Facebook discontinuance: discontinuance as a temporal settlement of the constant interplay between disturbance and coping. Quality & Quantity. 2015 7 1;49(4):1531–48.

[10] Schoenebeck SY. Giving up Twitter for Lent: how and why we take breaks from social media. In Proceedings of the SIGCHI Conference on Human Factors in Computing Systems 2014 Apr 26 (pp. 773–782). ACM.

[11] York C, Turcotte J. Vacationing from facebook: Adoption, temporary discontinuance, and readoption of an innovation. Communication Research Reports. 2015 1 2;32(1):54–62.

[12] Diener E. Assessing subjective well-being: Progress and opportunities. Social indicators research. 1994 2 1;31(2):103–57.

[13] Kross E, Verduyn P, Demiralp E, Park J, Lee DS, Lin N, Shablack H, Jonides J, Ybarra O. Facebook use predicts declines in SWB in young adults. PloS one. 2013 8 14;8(8):e69841 10.1371/journal.pone.0069841 23967061 PMC3743827

[14] Verduyn P, Ybarra O, Résibois M, Jonides J, Kross E. Do social network sites enhance or undermine subjective well‐being? A critical review. Social Issues and Policy Review. 2017 1 1;11(1):274–302. 10.1111/sipr.12033

[15] Gerson J, Plagnol AC, Corr PJ. Passive and Active Facebook Use Measure (PAUM): Validation and relationship to the Reinforcement Sensitivity Theory. Personality and Individual Differences. 2017 10 15;117:81–90.

[16] Burke M, Marlow C, Lento T. Social network activity and social well-being. In Proceedings of the SIGCHI conference on human factors in computing systems 2010 Apr 10 (pp. 1909–1912). ACM.

[17] Vigil TR, Wu HD. Facebook users’ engagement and perceived life satisfaction. Media and Communication. 2015 7 20;3(1):5–16. 10.17645/mac.v3i1.199

[18] Festinger L. A theory of social comparison processes. Human relations. 1954 5;7(2):117–40.

[19] Feinstein BA, Hershenberg R, Bhatia V, Latack JA, Meuwly N, Davila J. Negative social comparison on Facebook and depressive symptoms: Rumination as a mechanism. Psychology of Popular Media Culture. 2013 7;2(3):161.

[20] Ding Q, Zhang YX, Wei H, Huang F, Zhou ZK. Passive social network site use and SWB among Chinese university students: A moderated mediation model of envy and gender. Personality and Individual Differences. 2017 7 15;113:142–6.

[21] Chen W, Fan CY, Liu QX, Zhou ZK, Xie XC. Passive social network site use and subjective well-being: A moderated mediation model. Computers in Human Behavior. 2016 11 1;64:507–14.

[22] Wang JL, Wang HZ, Gaskin J, Hawk S. The mediating roles of upward social comparison and self-esteem and the moderating role of social comparison orientation in the association between social networking site usage and subjective well-being. Frontiers in psychology. 2017 5 11;8:771 10.3389/fpsyg.2017.00771 28553256 PMC5425586

[23] Appel H, Crusius J, Gerlach AL. Social comparison, envy, and depression on Facebook: A study looking at the effects of high comparison standards on depressed individuals. Journal of Social and Clinical Psychology. 2015 4;34(4):277–89

[24] Hinsch C, Sheldon KM. The impact of frequent social Internet consumption: Increased procrastination and lower life satisfaction. Journal of Consumer Behaviour. 2013 11;12(6):496–505.

[25] Tromholt M. The Facebook experiment: Quitting Facebook leads to higher levels of well-being. Cyberpsychology, behavior, and social networking. 2016 11 1;19(11):661–6. 10.1089/cyber.2016.0259 27831756

[26] Vanman EJ, Baker R, Tobin SJ. The burden of online friends: the effects of giving up Facebook on stress and well-being. The Journal of social psychology. 2018 7 4;158(4):496–507. 10.1080/00224545.2018.1453467 29558267

[27] McCambridge J, De Bruin M, Witton J. The effects of demand characteristics on research participant behaviours in non-laboratory settings: a systematic review. PloS one. 2012 6 19;7(6):e39116 10.1371/journal.pone.0039116 22723942 PMC3378517

[28] Distribution of Instagram users worldwide as of January 2018, by age group. January 2018. [cited 2018 Oct 02]. Available from: https://www.statista.com/statistics/325587/instagram-global-age-group/

[29] Most popular social networks worldwide, ranked by number of active users. October 2018. [cited 2018 Oct 02]. Available from: https://www.statista.com/statistics/272014/global-social-networks-ranked-by-number-of-users/

[30] Facebook Company info. Palo Alto, CA: Facebook. Retrieved from http://newsroom.fb.com/company-info/ (2018).

[31] Instagram. About us. Retrieved from https://www.instagram.com/about/us/ 14TH September, 2018

[32] Leading countries based on number of Facebook users. October 2018. [cited 2018 Oct 02]. Available from: https://www.statista.com/statistics/268136/top-15-countries-based-on-number-of-facebook-users/

[33] Leading countries based on number of Instagram users. October 2018. [cited 2018 Oct 02]. Available from: https://www.statista.com/statistics/578364/countries-with-most-instagram-users/

[34] Ritsner M, Kurs R, Gibel A, Ratner Y, Endicott J. Validity of an abbreviated quality of life enjoyment and satisfaction questionnaire (Q-LES-Q-18) for schizophrenia, schizoaffective, and mood disorder patients. Quality of Life Research. 2005 9 1;14(7):1693–703. 16119181 10.1007/s11136-005-2816-9

[35] Watson D, Clark LA, Tellegen A. Development and validation of brief measures of positive and negative affect: the PANAS scales. Journal of personality and social psychology. 1988 6;54(6):1063 3397865 10.1037//0022-3514.54.6.1063

[36] Pagani M, Hofacker CF, Goldsmith RE. The influence of personality on active and passive use of social networking sites. Psychology & Marketing. 2011 5;28(5):441–56.

[37] Hormes JM, Kearns B, Timko CA. Craving Facebook? Behavioral addiction to online social networking and its association with emotion regulation deficits. Addiction. 2014 12;109(12):2079–88. 10.1111/add.12713 25170590

[38] Tandoc EC, Ferrucci P, Duffy M. Facebook use, envy, and depression among college students: Is facebooking depressing?. Computers in Human Behavior. 2015 2 28;43:139–46. 10.1016/j.chb.2014.10.053

